# Exploring the Determinants and Correlates of Health-Enhancing Physical Activity of Adults in Eastern Poland

**DOI:** 10.3390/s25185665

**Published:** 2025-09-11

**Authors:** Marian J. Stelmach, Joanna Baj-Korpak, Ewelina Niźnikowska, Barbara Bergier, Michał Bergier, Dorota Tomczyszyn, Adam Szepeluk, Paulo Rocha

**Affiliations:** 1 Faculty of Health Sciences, John Paul II University in Biala Podlaska, 21-500 Biała Podlaska, Poland; j.baj-korpak@dyd.akademiabialska.pl (J.B.-K.); e.niznikowska@dyd.akademiabialska.pl (E.N.); b.bergier@dyd.akademiabialska.pl (B.B.); m.bergier@dyd.akademiabialska.pl (M.B.); d.tomczyszyn@dyd.akademiabialska.pl (D.T.); a.szepeluk@dyd.akademiabialska.pl (A.S.); 2 Portuguese Institute of Sports and Youth (IPDJ), 1990-100 Lisbon, Portugal; paulo.rocha@ipdj.pt

**Keywords:** accelerometry, adults, eastern Poland, health-enhancing physical activity, socio-demographic factors, health status

## Abstract

**Highlights:**

**What are the main findings?**

- Median HEPA (MVPA, VPA, PA10+) was below WHO recommendations; >70% of participants did not meet the guidelines.
- Self-rated health showed small-to-moderate positive associations with MVPA/VPA/PA10+, while BMI and waist circumference were weakly negatively associated with MVPA/VPA; MVPA and VPA declined with age; singles, white-collar workers, and students were more active than the unemployed; no differences by urban–rural residence or housing type.

**What is the implication of the main finding?**

- Prioritize older and unemployed adults and those with poorer self-rated health/higher adiposity; use practical strategies to accumulate MVPA/VPA and brief screening via self-rated health.
- Implement population-wide interventions across community, primary care, and workplaces/universities; future longitudinal/experimental studies should establish directionality and refine targeting.

**Abstract:**

In Poland—especially in the less developed eastern regions—the level of health-enhancing physical activity (HEPA) remains below the WHO recommendations, and its determinants are not yet fully understood. The study was conducted as part of the international EUPASMOS PLUS project on a sample of 173 adult individuals living in eastern Poland. Physical activity was measured using accelerometers worn continuously for seven days (24/7). The duration of moderate and vigorous physical activity as well as episodes of physical activity lasting at least 10 min were analyzed. The median daily MVPA time was 50 min, and the median VPA time only 10 s, both below WHO recommendations of 150 min/week of MVPA or 75 min/week of VPA. Overall, more than 70% of participants did not meet the recommended levels. The level of HEPA was found to be below WHO recommendations, especially among men, individuals over 50 years old, and those who were professionally inactive. Higher physical activity levels were recorded among women and younger participants. Significant correlations were found between HEPA level and self-rated health status (ρ = 0.28–0.38, *p* < 0.001), as well as body mass index and waist circumference (ρ ≈ −0.20 to −0.30, *p* < 0.01). Although statistically significant, the effect sizes were small to moderate, indicating limited explanatory power. Unemployment negatively affected MVPA and VPA levels, while household size positively correlated with physical activity. Interventions promoting HEPA should consider demographic and regional diversity, with particular focus on less active groups such as older adults and the unemployed. It is also necessary to develop new screening tools aimed at easy and quick diagnosis of social groups that should be targeted by HEPA promotion strategies.

## 1. Introduction

Numerous studies have demonstrated that health-enhancing physical activity (HEPA) reduces the risk of many chronic non-communicable diseases (NCDs) [1,2] and is associated with improved well-being [3]. It represents a scientifically proven foundation of a healthy lifestyle, bringing benefits to both physical and mental health [4]. Currently, a vast body of scientific evidence indicates that HEPA levels primarily depend on several specific factors, as shown in the results of numerous studies and meta-analyses. A review of the literature published before 2017 revealed that among the many factors influencing physical activity, only some—related to individual and environmental characteristics—show statistically significant correlations [5]. Most authors point to demographic characteristics such as age, gender, and socio-economic status as the main determinants [6]. Among the environmental factors significantly associated with physical activity patterns are access to green spaces and recreational sports infrastructure, as well as a supportive social environment [7]. Despite the proven health benefits, adherence to HEPA recommendations still falls short of the recommended standards, especially among women, youth, older adults, and residents of most high-income countries. This indicates the still underutilized health-promoting potential of regular physical activity [1,8,9].

Therefore, the promotion of HEPA requires comprehensive strategies that take into account a variety of contributing factors [10,11,12]. Moreover, conducting further research aimed at confirming or ruling out the influence of specific factors on HEPA levels—especially within populations where detailed studies have not yet been carried out—seems fully justified.

Systemic, multilevel determinants of physical activity, with a focus on HEPA, are essential in research conducted in populations and regions with limited research evidence, such as eastern Poland with its socio-economic challenges. The ecological model of physical activity proposed by Sallis et al. [13] is a widely adopted paradigm in such studies. It conceptualizes physical activity as the outcome of dynamic interactions across multiple levels: intrapersonal (e.g., demographics, biological, family situations), interpersonal (e.g., partners for activities), organizational (e.g., workplace infrastructure), environmental (e.g., availability of green spaces), and policy (e.g., public recreation investments).

In this model, socio-demographic characteristics act both directly as beneficial factors and indirectly as confounding factors that shape exposure and susceptibility to overarching socio-environmental opportunities and constraints. Meta-analyses and international comparative studies—including the Lancet series on the correlation between physical activity [14] and the results presented in the extensive IPEN study—consistently indicate that, for example, urban form promotes locomotor activity and that access to recreational facilities and proactive spatial planning are associated with higher levels of health-enhancing physical activity, regardless of individual characteristics [15]. At the same time, socio-demographic patterns of activity vary across different domains (recreation, transportation, work, and household), which may mask inequalities when only total physical activity is considered [16]. Against this background, the context of eastern Poland—characterized by relative socio-economic underdevelopment—is theoretically an important environment for studying how socio-demographic and environmental factors correlate with health-enhancing physical activity measured by an accelerometer and whether environmental opportunities mitigate demographic differences in activity. In the Polish population, there is a limited body of scientific data regarding the factors of health-enhancing physical activity among adults, with most of this data originating from studies based on subjective methods [17,18,19]. The 2021 report by the Public Health Committee of the Polish Academy of Sciences [20] shows that as many as 82% of Poles over the age of 15 do not meet WHO recommendations for physical activity.

As demonstrated by Romanowska et al. [21], programs promoting physical activity in Poland at the national level, which assumed a 3.5% increase in health-promoting physical activity and aimed to bring the percentage of residents who never play sports or exercise in line with the EU average, have not fulfilled their role. A mid-term assessment indicates that, despite a slight improvement in the situation, Poland is still far from achieving these goals [22].

The study of the factors (correlates and determinants) of health-promoting physical activity among Poles, in accordance with the definitions proposed by Bauman and co-authors [14,23], conducted based on the socio-ecological theory of health behavior [24] using an objective measurement method, is an attempt to fill the existing research gap in this area in the Polish population.

Guided by an ecological approach that places physical activity behaviors at interrelated levels, this study sought answers to the following questions: (1) What is the level of health-enhancing physical activity among residents of the studied region in relation to WHO guidelines? (2) To what extent are health indicators related to each form of health-enhancing physical activity included in the study? (3) Which socio-demographic characteristics are most strongly correlated with HEPA levels in the forms operationalized in the study?

The results presented in this study are of particular value as they are based on an analysis of objective data collected using accelerometers. They make a significant contribution to the understanding of the determinants of health-enhancing physical activity (HEPA) among adult Poles living in eastern Poland—a region characterized by substantial socio-economic underdevelopment in comparison to both the central-western part of the country and the European Union as a whole.

## 2. Materials and Methods

### 2.1. Study Design

The study was conducted in 2019–2020 as part of the international EUPASMOS PLUS project (https://erasmus-plus.ec.europa.eu/projects/search/details/603328-EPP-1-2018-1-PT-SPO-SCP (accessed on 22 October 2024)).

On the first day, each participant was informed about the purpose and procedure of the study. After obtaining written informed consent, a questionnaire-based interview was conducted to collect basic socio-demographic data. Participants were then instructed on how to properly wear and use the accelerometer. All participants agreed to wear the accelerometer for seven consecutive days (24/7).

### 2.2. Participants

The study group consisted of 173 individuals aged 18 to 79 years, purposefully selected from ethnically homogeneous Polish citizens residing in Biała Podlaska County, located in the eastern part of the country. Participants were divided into subgroups of similar size, taking into account gender (female and male) and age categories: <35, 35–49, 50–64, and >64 years.

Inclusion criteria were as follows: (a) being at least 18 years of age, and (b) having no medical contraindications to engaging in health-enhancing physical activity. Exclusion criteria included the following: (a) lack of consent to participate, (b) age under 18, and (c) health conditions preventing participation in physical activity. Socio-demographic data were collected using the EHIS-wave 3 questionnaire (European Health Interview Survey) [25]. This provided information on the area of residence, type of housing, household size (number of persons living in the household), and marital status. In addition, respondents assessed their general health using a 5-point Likert scale.

All participants provided written informed consent prior to enrolment. Accelerometer wear-time was monitored, and compliance was additionally verified using self-reported activity diaries. No participants withdrew due to device-related discomfort, and no behavioral changes attributable to continuous tracking were reported. Data were anonymized, stored on secure servers, and processed in compliance with institutional ethics approval and EU GDPR regulations. Encryption procedures and protocols for data deletion after project completion were applied to safeguard participant privacy.

### 2.3. Anthropometric Measurements

During the first meeting, anthropometric measurements were performed on all study participants. These included the following: (a) height measurement using a SECA 213 stadiometer with an accuracy of 0.10 cm; (b) waist and hip circumference measured with a SECA 201 anthropometric tape, also accurate to 0.10 cm; and (c) body weight measured using a Tanita BC-418 scale (Tanita Corporation, Tokyo, Japan) with an accuracy of 0.01 kg. Based on these measurements, Body Mass Index (BMI) was calculated.

### 2.4. Accelerometer Measurements

**Figure 1:**
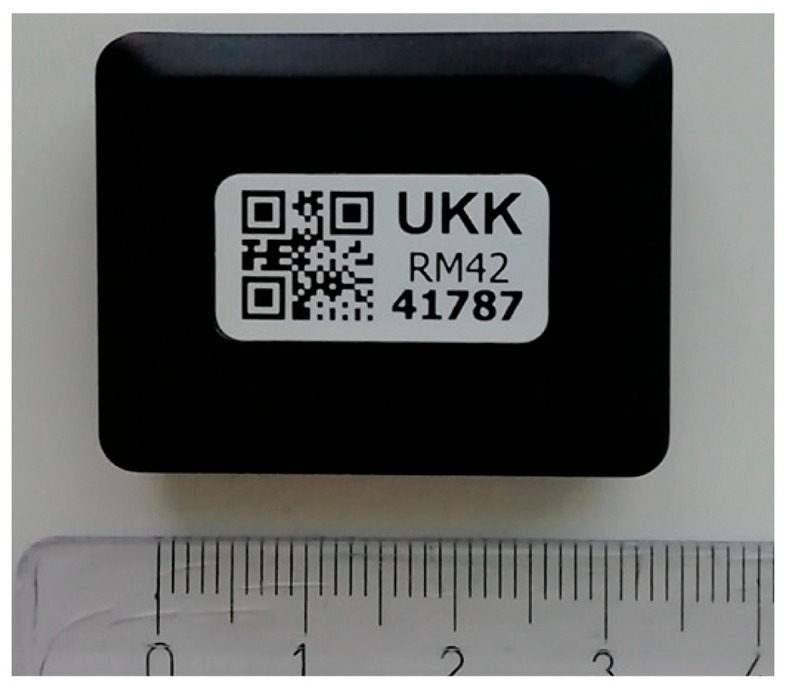
Triaxial accelerometer, model RM42, used in the study.

Physical activity was continuously monitored over a period of seven consecutive days (24 h per day) using a triaxial accelerometer, model RM42 (UKK, Tampere, Finland)—Figure 1.

**Figure 2:**
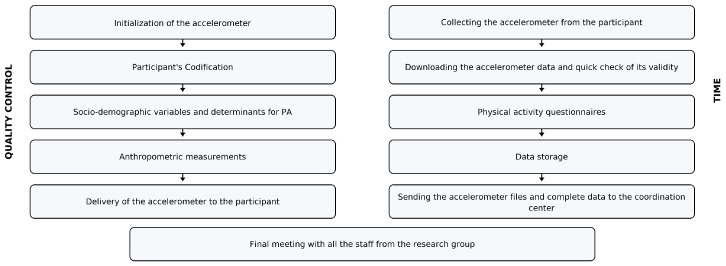
Harmonization process and quality control.

The device recorded acceleration data at a sampling frequency of 100 Hz with 13-bit analog-to-digital conversion within a range of ±16 g. The collected data were analyzed in non-overlapping 5 s epochs, with Mean Amplitude Deviation (MAD) calculated for each epoch [26]. During accelerometer initialization, the following parameters were set: (a) epoch length—6 s; (b) recording start time—immediately after activation; (c) interval between device initialization and participant wear—reduced to a minimum; (d) valid day/minimum number of days—>600 min of wear per day, ≥4 valid days including ≥1 weekend day; (e) non-wear definition—≥120 consecutive minutes of zero acceleration; (f) cut-points for physical activity—SB < 22.5 mg < LIPA < 91 mg < MPA < 414 mg < VPA; (g) missing data—only wear-time periods were analyzed (no imputation); (h) outliers/invalid records—flagged during quality control and excluded. The quality of the collected data was ensured thanks to the data harmonization structure (Figure 2).

**Figure 3:**
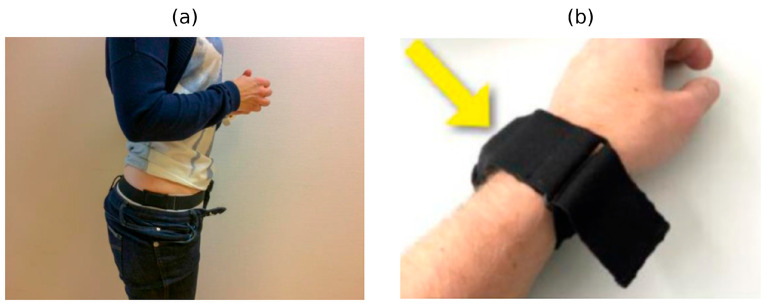
Accelerometer wear positions used for data collection: (**a**) hip placement during daytime—device attached at the waist/belt; (**b**) wrist placement during nighttime—device secured with a soft fabric wristband (yellow arrow) for comfortable sleep monitoring.

The accelerometer was secured with an elastic belt and a dedicated wrist strap. During the day, participants wore the device on the right side of the hip at the level of the iliac crest (Figure 3a), and during sleep, it was worn on the wrist of the non-dominant hand (Figure 3b).

Raw accelerometer data were transmitted to the UKK Institute for Health Promotion Research, where they were processed into numerical data suitable for statistical analysis. Variables used to characterize health-enhancing physical activity (HEPA) included vigorous physical activity (VPA), moderate-to-vigorous physical activity (MVPA), and physical activity sustained for more than 10 min (PA10+).

### 2.5. Statistical Analysis

Data collected through surveys and accelerometer measurements were statistically processed using IBM SPSS Statistics 29. Basic descriptive statistics were analyzed using the Shapiro–Wilk test, Mann–Whitney U test, and Spearman’s rho correlation analysis. A significance level of α = 0.05 was adopted for all analyses. Because HEPA variables deviated from normality (with VPA markedly right-skewed), we used rank-based non-parametric tests: Spearman’s ρ with 95% confidence intervals for associations, and Mann–Whitney U/Kruskal–Wallis with Dunn’s post hoc and Bonferroni adjustment for group comparisons; effect sizes are reported as η^2^. Ordinary least squares models for MVPA, VPA, and PA10+ included age and sex as a priori controls and a limited set of theoretically justified predictors to avoid overfitting. Multicollinearity (VIF < 2.0) and exploratory checks of non-linearity and first-order interactions were conducted; no material violations were observed. A significance level of α = 0.05 was adopted for all analyses. To examine differences between groups based on employment status, Dunn’s post hoc test was applied. Additionally, linear regression modeling was conducted to estimate the strength of influence of selected variables on the level of health-enhancing physical activity (HEPA).

## 3. Results

Analysis of the distribution of quantitative variables obtained in our study showed that all variables related to HEPA exhibited a right-skewed asymmetric distribution. For the VPA variable, the skewness value was 3.23, indicating significant asymmetry (Table 1). In the entire study cohort, the median health-enhancing physical activity (cumulative time of MVPA and VPA) as well as the median cumulative time of activity performed in sessions lasting longer than 10 min (PA10+) were below the levels recommended in the latest WHO guidelines [27].

### 3.1. Health Correlates of HEPA

**Figure 4:**
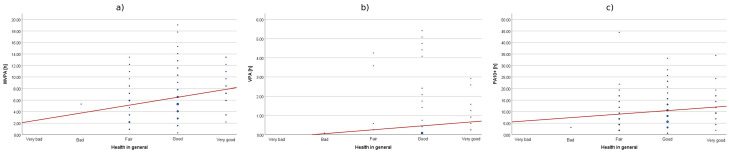
The correlation between subjective health status and cumulative time of MVPA (**a**), VPA (**b**), and PA10+ (**c**).

Among the health-related factors associated with HEPA levels, subjective health status and anthropometric indicators such as Body Mass Index (BMI) and waist circumference (WC) were considered. The analysis revealed statistically significant positive correlations between self-rated health status and levels of health-enhancing physical activity of varying intensities: moderate-to-vigorous physical activity (MVPA), vigorous physical activity (VPA), and cumulative activity performed in sessions lasting more than 10 min (PA10+). This indicates that as self-rated health status improved, both the cumulative time spent performing activities of different intensities and the duration of individual activity bouts increased. It should be emphasized, however, that these relationships are correlational in nature and do not allow for causal inferences. However, it is important to note that the effect size for vigorous physical activity was moderate (0.30 < ρ < 0.50), whereas in other cases it was small (ρ < 0.30). Although all correlations reached statistical significance, they should be interpreted with caution because the 95% CI indicates that the strength of this relationship may range from weak to moderate, which may limit the robustness of this finding. The associations between physical activity and health indicators, as well as the differences across demographic subgroups, were statistically significant but generally small to moderate in magnitude, suggesting that they explained only a limited share of the observed variability. The results are presented in Table 2 and Figure 4.

In the case of anthropometric health measures, statistically significant negative correlations were found between BMI and MVPA and VPA, as well as between WC and VPA (*p* < 0.001), and between WC and MVPA (*p* = 0.011). However, no significant correlations were observed between the anthropometric indicators and the duration of single physical activity sessions lasting more than 10 min (PA10+). The correlations between anthropometric indicators and physical activity levels were found to be weak, limiting their explanatory power. Given the cross-sectional design of this study and the relatively small sample size, the results should be interpreted as associations rather than evidence of causal relationships. Future longitudinal or experimental studies are needed to establish causality and explore temporal dynamics in these relationships.

### 3.2. Socio-Demographic Correlates of HEPA

#### 3.2.1. Gender and Age

Statistically significant differences in HEPA levels were also observed in groups divided by gender and age. For gender, significant differences were found for MVPA and PA10+ at *p* = 0.006 and 0.002, respectively, while for age groups, significant differences were noted for MVPA and VPA at *p* < 0.001. The effect size of gender differences was moderate to small, whereas in age groups it was moderate to large (Table 3).

Although statistically significant differences in HEPA levels were found when socio-demographic variables were considered, the magnitude of the effects indicates that the scale of these differences is limited. Furthermore, as the interaction effects between gender and age were not examined, the potential moderating influence of these variables remains unknown.

#### 3.2.2. Area of Residence and Type of Housing

Comparison of HEPA levels between groups categorized by place of residence (urban vs. rural) and type of housing (apartment vs. house) was conducted using the non-parametric Mann–Whitney U test, taking into account the statistically significant unequal sizes of the compared groups. The analysis did not reveal any statistically significant differences between the groups (Table 4). This indicates that, regardless of whether the participants lived in rural or urban areas, or in apartments or houses, they exhibited similar levels of health-enhancing physical activity.

#### 3.2.3. Household Size

**Figure 5:**
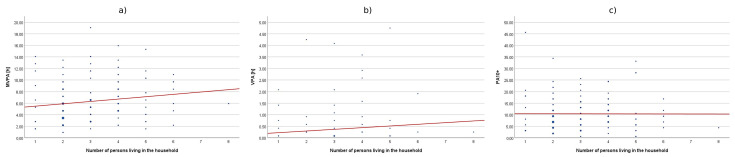
Correlations between household size and cumulative time of MVPA (**a**), VPA (**b**), and PA10+ (**c**).

Statistically significant positive correlations between HEPA levels were also observed in groups categorized by household size. As the number of household members increased, so did the cumulative time spent in moderate-to-vigorous physical activity (MVPA) and vigorous physical activity (VPA). However, no significant correlations were found regarding the duration of activity bouts exceeding 10 min (PA10+). The results are presented in Table 5 and illustrated in Figure 5.

#### 3.2.4. Marital Status

**Figure 6:**
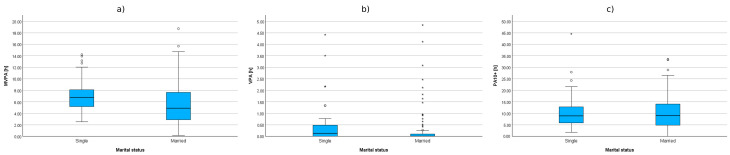
Cumulative time of MVPA (**a**), VPA (**b**), and PA10+ (**c**) in groups distinguished by marital status.

Another factor included in the correlation analyses was the participants’ marital status. Due to significantly unequal sizes of the groups of individuals in a relationship and those not in a relationship, the Mann–Whitney U test was applied. The analysis showed that individuals not in a relationship had significantly higher levels of moderate-to-vigorous physical activity (MVPA) and vigorous physical activity (VPA) compared to those in a relationship. The effect size for MVPA was small (η^2^ < 0.06), while for VPA it was moderate (0.06 < η^2^ < 0.14). Regarding physical activity sessions lasting 10 min or longer (PA10+), the differences were not statistically significant. Group differences by marital status were small to moderate implying modest practical significance despite statistical significance. The results are presented in Table 6 and illustrated in Figure 6.

#### 3.2.5. Employment Status

The final factor included in the correlation analyses was the employment status of the participants. The correlations between HEPA levels in groups distinguished by employment status were examined using the non-parametric Kruskal–Wallis test. The analysis revealed statistically significant differences between the compared groups in all three subcategories of health-enhancing physical activity. It is worth noting that in the case of MVPA, the observed effect size was moderate (0.06 < η^2^ < 0.14), while for VPA, it was large (η^2^ > 0.14). In contrast, for the cumulative time of physical activity performed in sessions lasting more than 10 min (PA10+), the effect size was small (η^2^ < 0.06).

To further examine the significance of differences between these groups, a post hoc Dunn’s test was conducted. The results of this test showed the following:

- White-collar workers, compared to non-working individuals, demonstrated a significantly longer MVPA time (Z = 4.25; *p* < 0.001), longer VPA time (Z = 5.80; *p* < 0.001), as well as longer cumulative time of physical activity performed in bouts lasting more than 10 min (Z = 2.21; *p* = 0.027);
- Students, in comparison to non-working individuals, also exhibited significantly longer MVPA time (Z = 3.80; *p* < 0.001) and VPA time (Z = 5.93; *p* < 0.001).

**Figure 7:**
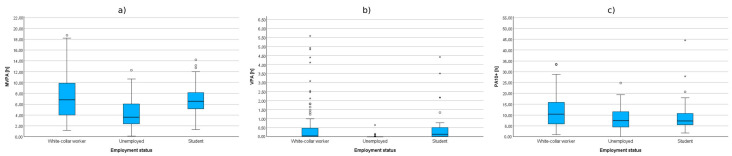
Cumulative time of MVPA (**a**), VPA (**b**), and PA10+ (**c**) in groups defined by employment status.

Detailed results are presented in Table 7 and Figure 7.

For employment status, effect sizes were moderate for MVPA (η^2^ = 0.11) and large for VPA (η^2^ = 0.25), pointing to a practically meaningful disparity in vigorous activity across groups, whereas the effect for PA10+ was small (η^2^ = 0.02). It is important to note that employment status may be confounded by other unmeasured variables such as income, education, or family responsibilities, which were not included in the current analysis.

### 3.3. Regression Models Explaining Health-Enhancing Physical Activity Time Based on Health-Related and Socio-Demographic Correlates

Before conducting the regression analyses, we examined the predictors for multicollinearity using variance inflation factor (VIF) values, which did not exceed the commonly accepted threshold (VIF < 2.0). Thus, multicollinearity was not considered problematic. Nevertheless, given the modest sample size and the number of predictors entered into the models, overfitting remains a potential limitation.

The linear regression analysis revealed that only the model predicting cumulative MVPA time was well-fitted to the data, explaining 11.6% of the variance in this variable. It was found that a statistically significant predictor of health-enhancing physical activity at a moderate-to-vigorous intensity (MVPA) was lack of employment, with a negative correlation. This indicates that within the studied cohort, being unemployed or having student status was associated with lower levels of MVPA. Additionally, the results showed that the models tested for VPA and PA10+ time were not well-fitted to the data, accounting for only 2.5% and less than 0.1% of the variance in these variables, respectively. Both unemployment and student status were also statistically significant predictors of vigorous physical activity time (VPA), again showing a negative correlation. The results of the regression modeling are presented in Table 8.

The very low explained variance (R^2^ < 3%) suggests that these models are poorly fitted to the data and that the predictors examined explain only a negligible portion of the variability in these outcomes. Therefore, these findings should be interpreted with particular caution.

Due to the cross-sectional nature of our study, we cannot interpret the regression results as evidence of causal relationships between socio-demographic or health factors and physical activity. However, the results obtained indicate directional relationships and may serve as a basis for longitudinal and/or experimental studies conducted in this population in the future.

## 4. Discussion

The presented results indicate that, among the analyzed factors, employment status—specifically the lack of professional activity (i.e., unemployment, retirement, or being a student)—was a statistically significant predictor negatively affecting the level of health-enhancing physical activity (HEPA) in the studied cohort. Other factors demonstrated correlations with HEPA levels but did not show statistically significant correlations with its subcategories, such as MVPA, VPA, and PA10+.

Our findings show that most participants did not meet the recommended levels of HEPA, thus failing to comply with the WHO guidelines in this regard. Target groups for interventions aimed at promoting increased physical activity (PA) should include men, individuals over the age of 50, and those not professionally active. This is because our results showed that women were more active than men, which contrasts with the outcomes of a similar study conducted in a demographically comparable German population [28], where women were statistically less likely than men to meet WHO recommendations—especially in terms of aerobic activity.

The study by Pardo et al. [9], conducted in the Catalonian population, indicates that recommended levels of HEPA are most commonly achieved by young adults and individuals living in medium-sized cities with normal body weight. Our findings did not reveal significant differences with regard to the area of residence or anthropometric indicators. HEPA levels also did not differ significantly when considering marital status. However, in the case of MVPA, our results were consistent with those of the International Prevalence Study from 20 countries [29], which reported an inverse correlation between age and the level of this type of activity. Our findings also support the recommendations formulated by Pardo et al. [9], emphasizing that middle-aged adults, individuals with obesity, and those not engaged in professional work should be particularly encouraged to increase their levels of HEPA.

The results obtained in this study provided valuable insights that may facilitate the design of future research on health-enhancing physical activity (HEPA) among the adult Polish population. The data showing weak to moderate correlations between self-rated health status and HEPA highlight the need to incorporate more detailed measures of both mental and physical health in future cohorts. In line with the suggestion by Bauman et al. [30] regarding comprehensive health assessments, such an approach may improve the accuracy of predictive models.

Our findings underscore the importance of demographic and regional diversity in research on the health aspects of HEPA, as well as the particular relevance of variables such as gender, age, and employment status—factors recognized as key determinants and correlates of physical activity. These results align with the conclusions of the long-term international IPEN study, which emphasized the critical role of demographic variables in developing physical activity models [15]. The lower HEPA levels observed among professionally inactive individuals in our study further confirm that health interventions and policies should prioritize the support and promotion of physical activity within these social groups [31].

Equally important is the methodological aspect of future research on HEPA. The skewed distributions of time spent in various categories of health-enhancing physical activity indicate the need to develop new measurement and analytical tools that better capture irregular HEPA patterns and contribute to more personalized health promotion strategies in the coming decades, especially in the context of activity monitoring technologies [32].

The analysis of data obtained in our study regarding subjective health status, anthropometric indicators, socio-demographic status, and their correlations with MVPA, VPA, and PA10+ provided empirical evidence supporting the existence of unconscious and hedonic psychological motivations for engaging in physical activity, as discussed by Rhodes et al. [33].

Although we were unable to establish a clear causal impact of the analyzed variables on HEPA levels due to the absence of a control group, our results confirm observations made by other researchers concerning barriers to physical activity [34].

Our results, based on accelerometer measurements, indicate that the majority of participants did not meet the WHO guidelines for health-enhancing physical activity, a pattern consistent with findings reported across Europe and in other contexts [35,36]. Although the effect sizes observed in our study were small to moderate, similar associations have been demonstrated in large-scale prospective studies, such as the UK Biobank [37], which showed a dose–response relationship between MVPA and mortality risk. These parallels emphasize that even relatively low levels of accelerometer-measured physical activity may have important public health implications, particularly in under-studied regions such as eastern Poland.

## 5. Advantages and Limitations

A strength of our study is the high quality of the data on which the analysis was based. The data were obtained through objective measurements using validated devices worn continuously for 7 consecutive days, 24 h a day. Most available research results conducted in the Polish population come from survey-based studies, which do not fully reflect the actual level of physical activity, and the determinants studied in this way may be unreliable [38]. This work also makes a significant contribution to the scientific literature by providing new data on the determinants and correlates of HEPA among adults living in eastern Poland, a region previously underexplored in this regard, as confirmed by the literature review conducted by Guthold et al. [39] on regional inequalities in physical activity. The identification of statistically significant correlations, such as the negative impact of unemployment on MVPA and VPA, as well as differences in activity between sexes and age groups, enriches the understanding of factors influencing public health in the context of global health challenges [27]. The importance of this study is further underscored by the fact that its results can serve as a reference point for regional and international comparisons, especially in the context of WHO recommendations [40]. Although some of the observed effects are modest, we believe they remain relevant, particularly given the lack of accelerometer-based evidence from Poland. The additional comparisons and public health implications now included in the manuscript strengthen the contribution of our study and clarify its significance despite the limitations.

The presented study also has certain limitations. First, it was an observational study, which prevents drawing conclusions about causality. Second, the study cohort consisted of individuals residing in a geographically limited area of eastern Poland, which restricts the ability to formulate generalizable conclusions.

## 6. Conclusions

In this accelerometer-based study of adults from eastern Poland, median levels of health-enhancing physical activity (MVPA, VPA, and PA10+) were below current WHO recommendations; over 70% of participants did not meet the guidelines. Self-rated health showed small-to-moderate positive associations with MVPA/VPA/PA10+, whereas BMI and waist circumference showed weak negative associations with MVPA/VPA. Socio-demographic patterns were consistent: MVPA and VPA declined with age, and single participants, white-collar workers, and students were more active than the unemployed. In multivariable regression, only MVPA was explained to a modest extent (adjusted R^2^ ≈ 0.12), with unemployment emerging as an independent negative predictor. No differences were observed by urban–rural residence or housing type.

These findings provide objective, region-specific evidence on physical-activity correlates in eastern Poland and point to two actionable priorities: (1) targeted promotion for older and unemployed adults and for those with poorer self-rated health and higher adiposity; and (2) population-wide delivery across settings (community, primary care, workplaces/universities), given the absence of residence/housing disparities.

Given the cross-sectional design and the limited fit of some models, further longitudinal or experimental accelerometer-based studies in this population are needed to establish directionality and refine intervention targeting.

In summary, the determinants and correlates of health-enhancing physical activity (HEPA) among adults are multifaceted and include individual, social, and environmental factors. Interventions aimed at promoting physical activity should adopt a comprehensive approach based on the socio-ecological model of physical activity [13], which addresses these various levels of influence. Future research should continue to explore the complex interactions between personal, social, and environmental factors, as well as geographical differences in physical activity patterns, to inform about the development of effective, equitable, and sustainable strategies for increasing physical activity participation among adults.

The key issue addressed in this study—the identification of determinants and correlates of HEPA in the adult Polish population—is important in the context of global health challenges related especially to the prevention of chronic non-communicable diseases [40].

## Figures and Tables

**Table 1:** Basic descriptive statistics of HEPA variables along with the Shapiro–Wilk test.

PA Variables	M	Mdn	SD	Sk.	Kurt.	Min.	Max.	W	*p*-Value
MVPA	0:54:17	0:50:01	0:31:33	0.81	0.47	0:01:09	2:40:30	0.95	<0.001 *
VPA	0:03:42	0:00:10	0:08:40	3.23	10.60	0:00:00	0:47:51	0.53	<0.001 *
PA10+	1:28:03	1:17:36	1:01:15	1.44	3.20	0:00:00	6:21:36	0.93	<0.001 *

Note. * Statistical significance.

**Table 2:** Correlations between subjective health status and cumulative time of MVPA, VPA, and PA10+.

PA Variables	Health in General		
	ρ	95% CI	*p*-Value
MVPA	0.28	0.14–0.43	<0.001 *
VPA	0.38	0.25–0.51	<0.001 *
PA10+	0.19	0.05–0.35	0.011 *

Note. * Statistical significance.

**Table 3:** Correlations between gender and age groups and cumulative time of MVPA, VPA, and PA10+.

PA Variables	Male (n = 102)			Female (n = 71)					
	Mean Rank	M	SD	Mean Rank	M	SD	Z	*p*-Value	η^2^
MVPA	77.04	0:48:24	0:28:47	101.31	1:02:44	0:33:33	−3.14	0.002 *	0.06 ^(m)^
VPA	82.19	0:03:20	0:08:57	93.92	0:04:14	0:08:17	−1.56	0.120	0.01
PA10+	78.29	1:15:45	0:49:08	99.51	1:45:43	1:12:06	−2.74	0.006 *	0.04 ^(s)^
	**Age**		**Mean rank**	**M**	**SD**	**H(3)**	***p-*** **Value**		**η^2^**
MVPA	18–34 (n = 53)		104.69	1:03:31	0:27:57	23.16	<0.001 *		0.12 ^(m)^
	35–49 (n = 41)		101.34	1:02:37	0:32:07				
	50–64 (n = 44)		73.74	0:48:33	0:33:53				
	65 and older (n = 35)		60.09	0:37:43	0:25:03				
VPA	18–34 (n = 53)		111.00	0:04:44	0:09:00	52.21	<0.001 *		0.29 ^(h)^
	35–49 (n = 41)		106.39	0:06:29	0:11:18				
	50–64 (n = 44)		76.48	0:02:40	0:07:58				
	65 and older (n = 35)		41.17	0:00:11	0:00:56				
PA10+	18–34 (n = 53)		88.45	1:31:12	1:05:38	4.35	0.226		<0.01
	35–49 (n = 41)		97.99	1:36:54	0:56:59				
	50–64 (n = 44)		85.17	1:28:54	1:07:11				
	65 and older (n = 35)		74.23	1:11:51	0:49:50				

Note. * Statistical significance, ^(s)^ small effect size, ^(m)^ moderate effect size, ^(h)^ large effect size.

**Table 4:** Correlations between groups categorized by area of residence and type of housing and cumulative time of MVPA, VPA, and PA10+.

PA Variables	Urban Areas (n = 135)			Rural Areas (n = 37)					
	Mean Rank	M	SD	Mean Rank	M	SD	Z	*p*-Value	η^2^
MVPA	86.58	6:20:18	3:46:06	86.22	6:09:10	3:17:20	−0.04	0.969	<0.01
VPA	85.77	0:26:50	1:01:45	89.16	0:23:04	0:58:27	−0.38	0.706	<0.01
PA10+	88.48	10:19:11	6:46:46	79.27	9:57:40	8:29:31	−1.00	0.319	<0.01
	**Apartment (n = 73)**			**House (n = 100)**					
	**Mean Rank**	**M**	**SD**	**Mean Rank**	**M**	**SD**	**Z**	***p-*** **Value**	**η^2^**
MVPA	94.62	6:57:47	3:58:20	81.44	5:52:23	3:23:51	−1.71	0.087	0.02
VPA	88.38	0:38:56	1:19:35	85.99	0:16:33	0:39:51	−0.32	0.750	<0.01
PA10+	89.69	10:17:39	5:59:25	85.04	10:15:28	7:54:56	−0.60	0.546	<0.01

**Table 5:** Correlations between household size and cumulative time of MVPA, VPA, and PA10+.

PA Variables	Household Size	
	ρ	*p*-Value
MVPA	0.20	0.018 *
VPA	0.28	0.001 *
PA10+	0.04	0.601

Note. * Statistical significance.

**Table 6:** Correlations between groups distinguished by marital status and cumulative time of MVPA, VPA, and PA10+.

PA Variables	Single (n = 50)			Married (n = 91)					
	Mean Rank	M	SD	Mean Rank	M	SD	Z	*p*-Value	η^2^
MVPA	84.22	7:10:18	2:58:30	63.74	5:51:21	3:49:23	−2.85	0.004 *	0.06
VPA	86.28	0:27:42	0:52:41	62.60	0:19:18	0:50:28	−3.37	<0.001 *	0.08
PA10+	72.68	10:41:03	7:42:45	70.08	10:15:06	7:23:11	−0.36	0.717	<0.01

Note. * Statistical significance.

**Table 7:** Correlations between groups defined by employment status and cumulative time of MVPA, VPA, and PA10+.

PA Variables	Employment Status	Mean Rank	M	SD	H(2)	*p*-Value	η^2^
MVPA	White-collar worker (n = 87)	94.32	7:09:34	4:00:07	20.96	<0.001 *	0.11
	Unemployed (n = 43)	55.55	4:14:36	2:46:56			
	Student (n = 39)	96.69	6:56:36	2:58:00			
VPA	White-collar worker (n = 87)	95.61	0:37:03	1:13:30	43.99	<0.001 *	0.25
	Unemployed (n = 43)	44.01	0:01:30	0:06:07			
	Student (n = 39)	106.53	0:30:58	0:58:07			
PA10+	White-collar worker (n = 87)	93.87	11:34:14	7:27:43	6.03	0.049 *	0.02
	Unemployed (n = 43)	73.70	8:26:08	5:31:13			
	Student (n = 39)	77.67	9:38:34	7:44:23			

Note. * Statistical significance.

**Table 8:** Regression models for health-enhancing physical activity determined by health-related and socio-demographic factors (n = 146).

PA Variables	Demographic Characteristics	B	SE	Beta	t	*p*-Value
MVPA	*F*(6;132) = 4.01; *p* = 0.001; *R*^2^*adj*. = 0.116					
	(Constant)	22,882.82	9287.16		2.46	0.015 *
	Marital status (0—single; 1—married)	−2534.32	2388.35	−0.09	−1.06	0.291
	Unemployed (0—no; 1—yes)	−7763.25	2945.41	−0.25	−2.64	0.009 *
	Household size	581.62	873.91	0.06	0.67	0.507
	Area of residence (0—urban areas; 1—rural areas)	−1224.02	2776.07	−0.04	−0.44	0.660
	Type of housing (0—apartment; 1—house)	−3477.59	2351.70	−0.13	−1.48	0.142
	Health in general	3203.80	1651.36	0.16	1.94	0.055
VPA	*F*(6;132) = 1.60; *p* = 0.152; *R*^2^*adj*. = 0.025					
	(Constant)	1752.54	2331.62		0.75	0.454
	Marital status (0—single; 1—married)	50.23	599.62	<0.01	0.08	0.933
	Unemployed (0—no; 1—yes)	−1603.46	739.47	−0.22	−2.17	0.032 *
	Household size	117.04	219.40	0.05	0.53	0.595
	Area of residence (0—urban areas; 1—rural areas)	307.48	696.96	0.04	0.44	0.660
	Type of housing (0—apartment; 1—house)	−939.16	590.42	−0.15	−1.59	0.114
	Health in general	89.06	414.59	0.02	0.21	0.830
PA10+	*F*(6;132) = 0.53; *p* = 0.783; *R*^2^*adj*. < 0.001					
	(Constant)	27,791.16	20,759.43		1.34	0.183
	Marital status (0—single; 1—married)	185.47	5338.63	<0.01	0.03	0.972
	Unemployed (0—no; 1—yes)	−6628.88	6583.82	−0.10	−1.01	0.316
	Household size	−770.88	1953.43	−0.04	−0.39	0.694
	Area of residence (0—urban areas; 1—rural areas)	−1838.44	6205.31	−0.03	−0.30	0.767
	Type of housing (0—apartment; 1—house)	1580.87	5256.72	0.03	0.30	0.764
	Health in general	3870.19	3691.27	0.10	1.05	0.296

Note. * Statistical significance.

## Data Availability

The data presented in this study are available on request from the corresponding author due to consortium ownership and contractual restriction under the EUPASMOS parthership, as well as ethical and GDPR obligations that prevent the public deposition.

## Abbreviations

The following abbreviations are used in this manuscript:

HEPA	Health-enhancing physical activity
NCDs	Non-communicable diseases
MVPA	Moderate-to-vigorous physical activity
VPA	Vigorous physical activity
PA10+	Physical activity sustained more than 10 min
MAD	Mean amplitude deviation
BMI	Body Mass Index
WC	Waist circumference
